# Regulation of Gag- and Env-Specific CD8^+^ T Cell Responses in ART-Naïve HIV-Infected Patients: Potential Implications for Individualized Immunotherapy

**DOI:** 10.1371/journal.pone.0153849

**Published:** 2016-04-29

**Authors:** Christian Prebensen, Andreas Lind, Anne-Ma Dyrhol-Riise, Dag Kvale

**Affiliations:** 1 Department of Infectious Diseases, Oslo University Hospital, Oslo, Norway; 2 Institute of Clinical Medicine, University of Oslo, Oslo, Norway; 3 K.G. Jebsen Inflammation Research Centre, University of Oslo, Oslo, Norway; Istituto Superiore di Sanita, ITALY

## Abstract

Strategies to develop a functional cure for HIV infection will likely require boosting of effector T cell responses to eliminate reactivated, latently infected cells. We have recently explored an assay for assessing antigen-specific regulation of T cell proliferation, which was related to clinical progression in untreated patients and to vaccine efficacy in two trials of therapeutic Gag-based vaccines. We here expand the same assay to further investigate regulation mediated by various inhibitory pathways. Peripheral blood mononuclear cells from 26 asymptomatic HIV-infected, antiretroviral therapy-naïve patients were stimulated with Gag and Env overlapping peptide panels for 5 days. Monoclonal antibodies (mAbs) blocking inhibitory mediators interleukin (IL) 10, transforming growth factor (TGF) β, programmed death ligand (PD–L) 1 and herpes virus entry mediator (HVEM) were added to parallel cultures. Functional T cell regulation (FTR) was defined as the difference in proliferation between stimulated cultures with and without blocking mAbs. FTR was detected in 54% of patients. Blockade of IL-10/PD-L1 and IL10/TGF-β detected all cases with Gag- and Env-associated FTR, respectively. In accordance with previous findings, isolated Env FTR was associated with higher plasma HIV RNA and lower CD4 counts, while patients with both Gag and Env FTR also had higher Gag- and Env-specific proliferative CD8^+^ T cell responses. There was no association between FTR and frequencies of activated regulatory T cells. In conclusion, we observed substantial heterogeneity in FTR between patients, inhibitory pathways and HIV antigens. FTR may help to individualize immunomodulation and warrants further assessment in clinical immunotherapy trials.

## Introduction

Effective HIV-specific cytotoxic T lymphocyte (CTL) responses are central to immune control of HIV infection [1,2]. CD8^+^ T cell responses against HIV emerge during the course of acute infection, concurrently with falling plasma viremia [3,4]. The small minority of patients who naturally control HIV infection maintain highly effective HIV-specific CTL responses over time, exhibiting both polyfunctionality and potent HIV-suppressive effects [5,6]. On the other hand, most patients chronically infected with HIV progressively lose HIV-specific CD8^+^ T cell responses [7], through reduced CD4^+^ T cell help [8], clonal T cell loss [9], immune exhaustion [10] and other negative regulatory mechanisms. Importantly, these defects in HIV-specific T cell immunity are not fully restored by antiretroviral therapy (ART) [11,12].

Despite its ability to durably suppress HIV replication, ART does not eradicate the latent viral reservoir, and lifelong therapy is necessary to avoid rapid viral rebound [13]. This has sparked efforts to develop therapeutic strategies able to establish durable viral control in the absence of ART, a so-called functional cure [14,15]. Many of these approaches will require an induction or boosting of the patients’ impaired HIV-specific CTL function in order to eliminate reactivated, latently infected cells [16] and maintain viral control. This may be attained by therapeutic vaccination or other immunomodulatory therapy.

Antigen-induced T cell activation and proliferation are subject to negative regulation through a variety of signalling pathways, including the anti-inflammatory cytokines interleukin (IL) 10 and transforming growth factor (TGF) β as well as negative co-signalling molecules programmed death (PD) 1 and CD160. We have recently explored an assay for assessing negative regulation of HIV-specific T cell function mediated by IL-10 and TGF-β. This regulation parameter was associated with clinical progression in untreated HIV infection [17]. Moreover, pre-existing and evolving regulation of HIV vaccine-specific CD8^+^ T cell responses coincided with low final responses against therapeutic Gag peptide-vaccines in ART-treated patients [18,19]. Thus, such an approach of assessing antigen-induced T cell regulation may prove clinically useful and these data suggest that regulation should be taken into account when considering patients for immunomodulatory therapy as part of a functional cure. In addition, quantifying the contribution of various pathways in suppressing T cell function may allow individually tailored interventions directed at these mechanisms [20–23].

The aim of this study was to further explore mechanisms of functional T cell regulation (FTR) of CD8^+^ T cell responses against HIV Gag and Env antigens, mediated by not only IL-10 and TGF-β, but also PD-1/PD-L1 and CD160/HVEM pathways. We observed substantial heterogeneity in FTR between patients, inhibitory pathways and HIV antigens, and an apparent detrimental effect on clinical parameters of isolated Env-related FTR.

## Methods

### Study participants

Twenty-six asymptomatic HIV-1 seropositive patients from the Department of Infectious Diseases, University Oslo Hospital were included in the study. All included patients were above 18 years of age, ART-naïve and none fulfilled the criteria of elite or viremic controllers. No patients were co-infected with hepatitis B or C virus and none had symptoms or findings of intercurrent or opportunistic infections. CD4^+^ and CD8^+^ T cell counts and plasma HIV RNA were determined by routine clinical assays. Clinical characteristics are shown in Table 1.

**Table 1:** Clinical characteristics (n = 26).

**Age (years)**	42.5 (40.0–49.0) *
**Gender (male/female)**	23/3
**Time since HIV diagnosis (years)**	2.0 (1.0–3.9)
**CD4^+^ T cell counts (cells/uL)**	416 (337–514)
**CD8^+^ T cell counts (cells/uL)**	1 325 (1 054–1 611)
**Plasma HIV RNA (copies/mL)**	34 500 (10 000–81 000)

*: Data given as median (lower—upper quartile)

The study was approved by The Norwegian South-Eastern Regional Committee for Medical and Health Research Ethics. Written, informed consent was obtained from all study participants.

### Samples

Peripheral blood mononuclear cells (PBMC) were isolated using Vacutainer Cell Preparation Tubes (Becton Dickinson (BD), NJ, USA). Cells were frozen in RPMI containing 40% FCS and 10% DMSO and stored at -145°C. CD4^+^ and CD8^+^ T cell counts and plasma HIV RNA loads were determined by routine clinical assays.

### Proliferation/Regulation assays

Cells were thawed, washed and reconstituted in AIM V serum-free medium (Life Technologies, Oslo, Norway) containing 0.1% human serum albumin. After an overnight rest, cells were pulse-labelled with *carboxyfluorescein diacetate succinimidyl ester* (CFSE, Life Technologies) at a concentration of 2 μM for 5 minutes. The following blocking antibodies were added to parallel culture wells at a final concentration of 10 μg/mL: anti-IL-10 (R&D Systems, MN, USA; clone 23738), anti-TGF-β (R&D; clone 1D11), anti-PD-L1 (eBioscience, CA, USA; clone MIH1), and anti-HVEM (R&D; clone 94801).

After a 30 minute incubation, cultures were stimulated with either Gag or Env 15-mer overlapping peptide panels (NIH AIDS Research and Reference Reagent Program, MD, USA) at a final concentration of 2 μg/mL/peptide. Staphylococcal Enterotoxin B (SEB, Sigma-Aldrich, MO, USA) at a final concentration of 0.5 μg/mL was used as a positive control.

Cells were cultured at 37°C in 5% CO_2_ for 5 days, harvested, and stained with the following fluorochrome-conjugated antibodies: CD3 V450, CD8 APC-H7, HLA-DR BV605, CD45RA APC (all BD) and CD25 PE (Biolegend). 7-aminoactinomycin D (7-AAD, BD) was added for dead cell exclusion. Flow cytometry data were acquired on a BD FACS Canto II with BD Diva 6.1 software, and analyzed in FlowJo X (FlowJo LLC, OR, USA).

### Gating, readouts and parameter definitions

**Fig 1:**
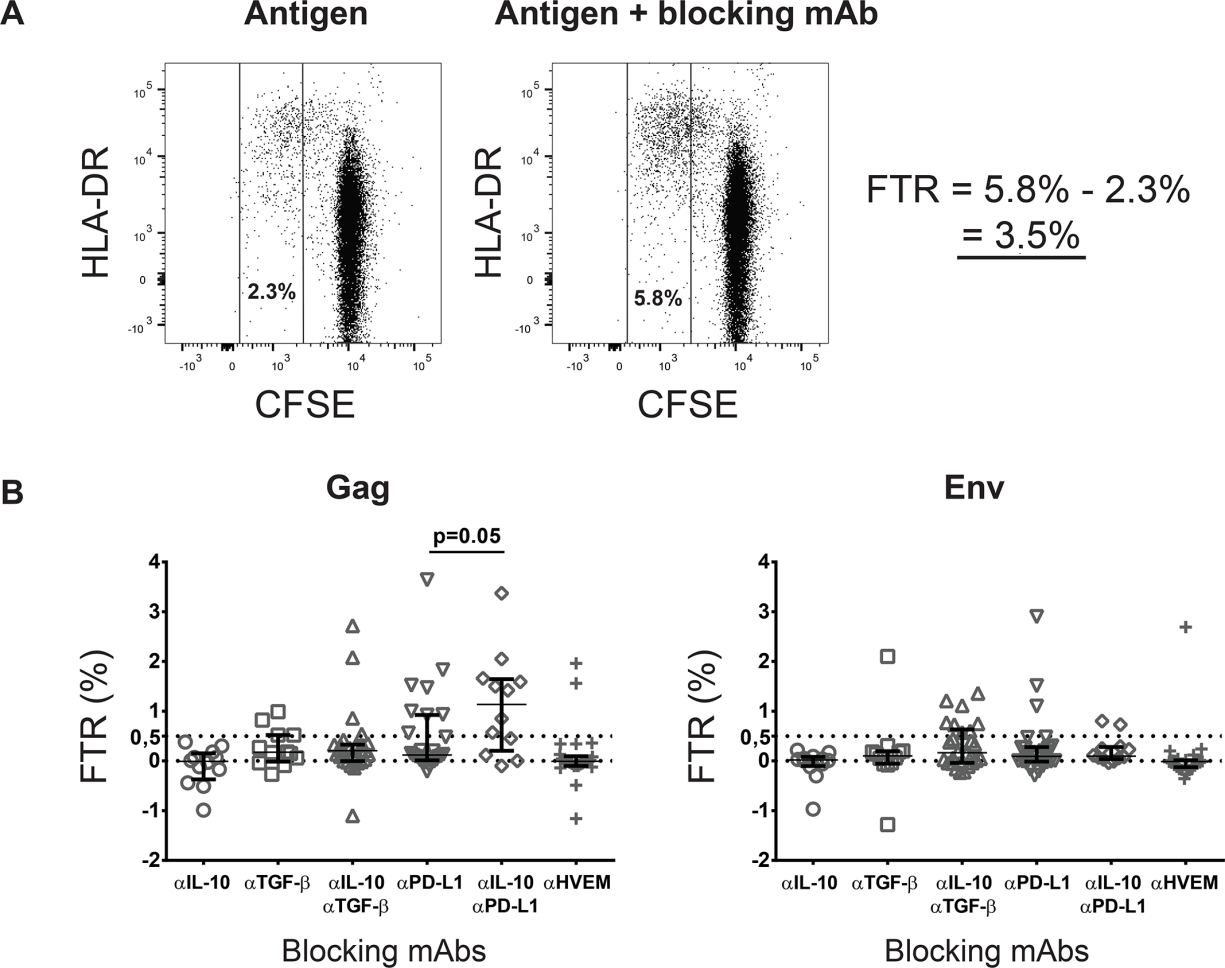
Functional T cell regulation (FTR) of CD8^+^ T cells from HIV-infected patients. (A) FTR as the difference between proliferating (CFSE^dim^) CD8^+^ T cells, in HIV antigen-stimulated cultures with and without blocking antibodies. Proliferating cells also express the activation marker HLA-DR. (B) Gag and Env FTR, assessed by single or dual blockade of inhibitory pathways. Median and upper/lower quartiles indicated. FTR defined as an increase in proliferating cells above a threshold of 0.5% (indicated by upper dashed line). P-value derived from paired test.

Antigen-specific, proliferated CD8^+^ T cells were defined by CFSE fluorescence between the second and sixth generation of CFSE^dim^ cells (Fig 1A). The first generation was omitted based on the assumption that these late proliferating cells more likely represent unspecific bystander proliferation. Activated CD8^+^ T cells were defined by co-expression of CD25 and HLA-DR. Activated regulatory T cells (aTregs) were defined by the phenotype CD3^+^CD8^-^CD25^hi^CD45RA^-^ [24]. In the absence of discrete cell populations, gating cut-offs were determined by the Fluorescence Minus One (FMO) method. Examples of all gates are given in S1 Fig.

Proliferative responses were defined as the difference in CFSE^dim^ CD8^+^ T cell fractions between antigen-stimulated and unstimulated cultures. Functional T cell regulation (FTR) was defined as the difference in the percentage of CFSE^dim^ CD8^+^ T cells between stimulated cultures with and without blocking antibodies, respectively (Fig 1A). In addition, a lower threshold of 0.5% was set to signify FTR beyond potential intra-assay variability, as we observed occasional negative FTR values between 0 and -0.5% (excluding a few outliers). FTR values >0.5% were consistently found on repeated analysis of selected samples.

### Statistics

All statistical methods used were non-parametric. Comparisons between groups were assessed by Kruskal-Wallis and Mann-Whitney U tests. Correlations between parameters were assessed by Spearman rank sum tests. Paired data was assessed by Wilcoxon matched pair tests. Analyses were performed in SPSS v. 21 (IBM Corp. Armonk, NY) and Graphpad Prism 6 (Graphpad Software, La Jolla, CA). P-values below 0.05 were considered statistically significant.

## Results

### Heterogeneity in Gag and Env regulation

Samples from all patients were first assessed for CD8^+^ FTR mediated by IL-10/TGF-β, the PD-1/PD-L1 and the CD160/HVEM pathways, respectively. Overall, 14 patients (54%) had detectable FTR. In a subgroup of 12 patients who had quantifiable Gag and/or Env FTR detected by IL10/TGF-β dual blockade, we repeated the experiments with single blockade of IL-10 and TGF-β, to quantify the individual regulatory contribution of these two cytokines. In addition, we investigated the FTR effects of PD-L1, IL-10/PD-L1 and HVEM blockade in Gag and Env-stimulated cultures, respectively (Fig 1B).

We observed considerable heterogeneity, not only between Gag and Env FTR, but also between the regulatory effects of the different inhibitory pathways (Fig 1B). For example, we found no Gag or Env FTR mediated by IL-10 alone, while four patients had Gag FTR mediated by TGF-β alone, one of which also had Env FTR mediated by TGF-β. However, Env FTR assessed by IL-10/TGF-β dual blockade was observed in eight patients, suggesting additive or synergistic effects of the two cytokines on Env responses. For Gag FTR, an additive effect was obtained by blocking IL-10 in combination with PD-L1 (p = 0.05), but no such synergy was found for Env FTR (Fig 1B).

Blockade of the CD160 ligand HVEM identified only two patients with Gag FTR and another patient with Env FTR. No clear associations were found between HVEM FTR assessments and FTR obtained by the other inhibitory pathways.

Taken together, substantial inter-individual differences were noted for FTR in relation to both signalling pathways and HIV antigens.

### Different blocking conditions best identify patients with Gag and Env FTR

**Fig 2:**
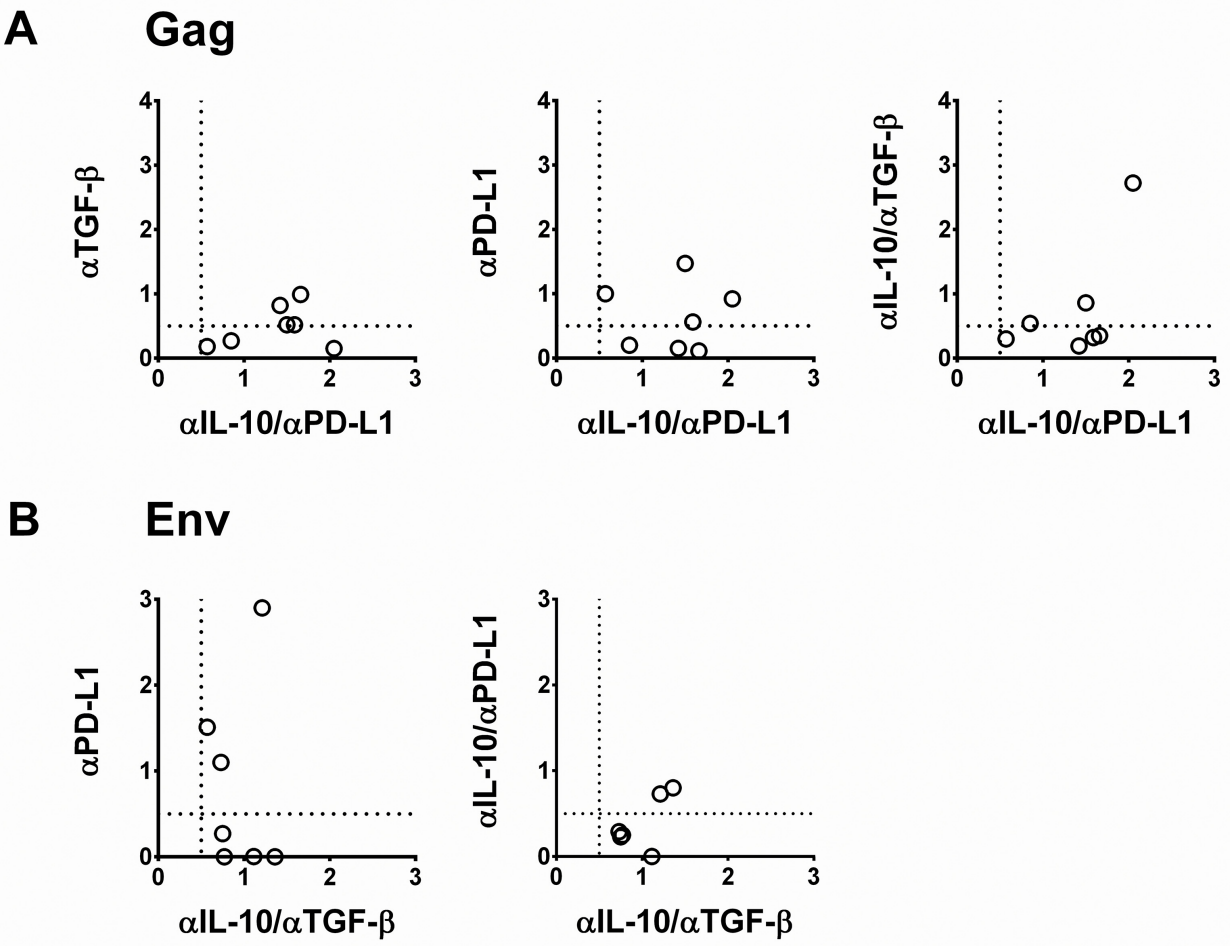
Gag and Env FTR is best identified by different combinations of blocking mAbs. The relationship between FTR mediated by various pathways in (A) Gag- and (B) Env-stimulated cultures. Plots include patients with detectable FTR on one or both axes. Only dual blockade of IL-10/PD-L1 (x-axis) identifies all patients with Gag FTR whereas dual blockade of IL-10/TGF-β (x-axis) identifies all patients with Env FTR. FTR defined as an increase in proliferating cells above a threshold of 0.5% (dashed lines).

Because we hypothesized that HIV antigen-specific FTR is important to quantify in a clinical immunotherapy setting, we next selected the assay conditions which revealed the highest number of patients with quantifiable Gag and Env FTR, respectively. Notably, dual blockade of IL-10/PD-L1 revealed all patients with Gag FTR, whereas combined blockade of IL-10/TGF-β identified all patients with Env FTR (Fig 2).

The heterogeneity among patients who had detectable FTR was once again exemplified by there being no significant concordance between Gag and Env FTR mediated by the same signalling pathways (data not shown).

### Regulation associated with viremia and CD4 counts

**Fig 3:**
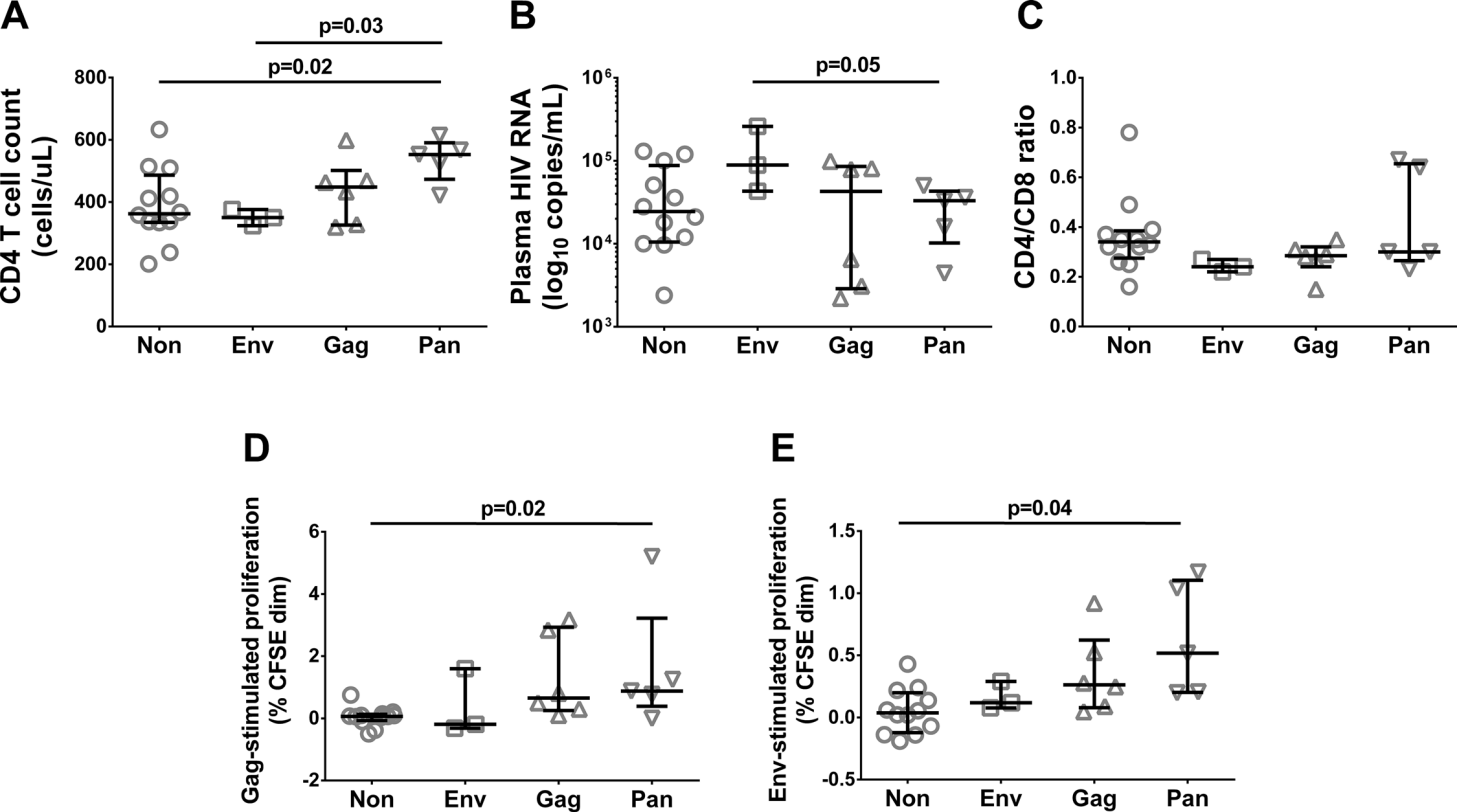
Clinical and immunological variables of regulator subgroups. (A) CD4 count, (B) Plasma HIV RNA, (C) CD4/CD8 ratio, (D) Gag-stimulated proliferation and (E) Env-stimulated proliferation, by regulator subgroup. Median and upper/lower quartiles indicated. P-values derived from unpaired tests.

Despite the heterogeneity in FTR by signal pathways and antigens, patients were tentatively grouped on the basis of whether they exhibited FTR associated with Gag only (Gag-regulators, n = 6), Env only (Env-regulators, n = 3), both Gag and Env (Pan-regulators, n = 5), or neither Gag nor Env (Non-regulators, n = 12). There were no differences in age or time since HIV diagnosis between the regulator subgroups. While the paucity of Env-regulators hampered statistical analysis, this group nevertheless had lower CD4 counts (p = 0.03) and higher viral loads (p = 0.05) than Pan-regulators (Fig 3A and 3B). All Env regulators had a low CD4/CD8 ratio, although there were no differences between groups (Fig 3C). Pan-regulators, on the other hand, had higher CD4 counts (p = 0.02) and higher both Gag- and Env-specific proliferative responses than Non-regulators (p = 0.02 and p = 0.04, respectively) (Fig 3D and 3E).

**Fig 4:**
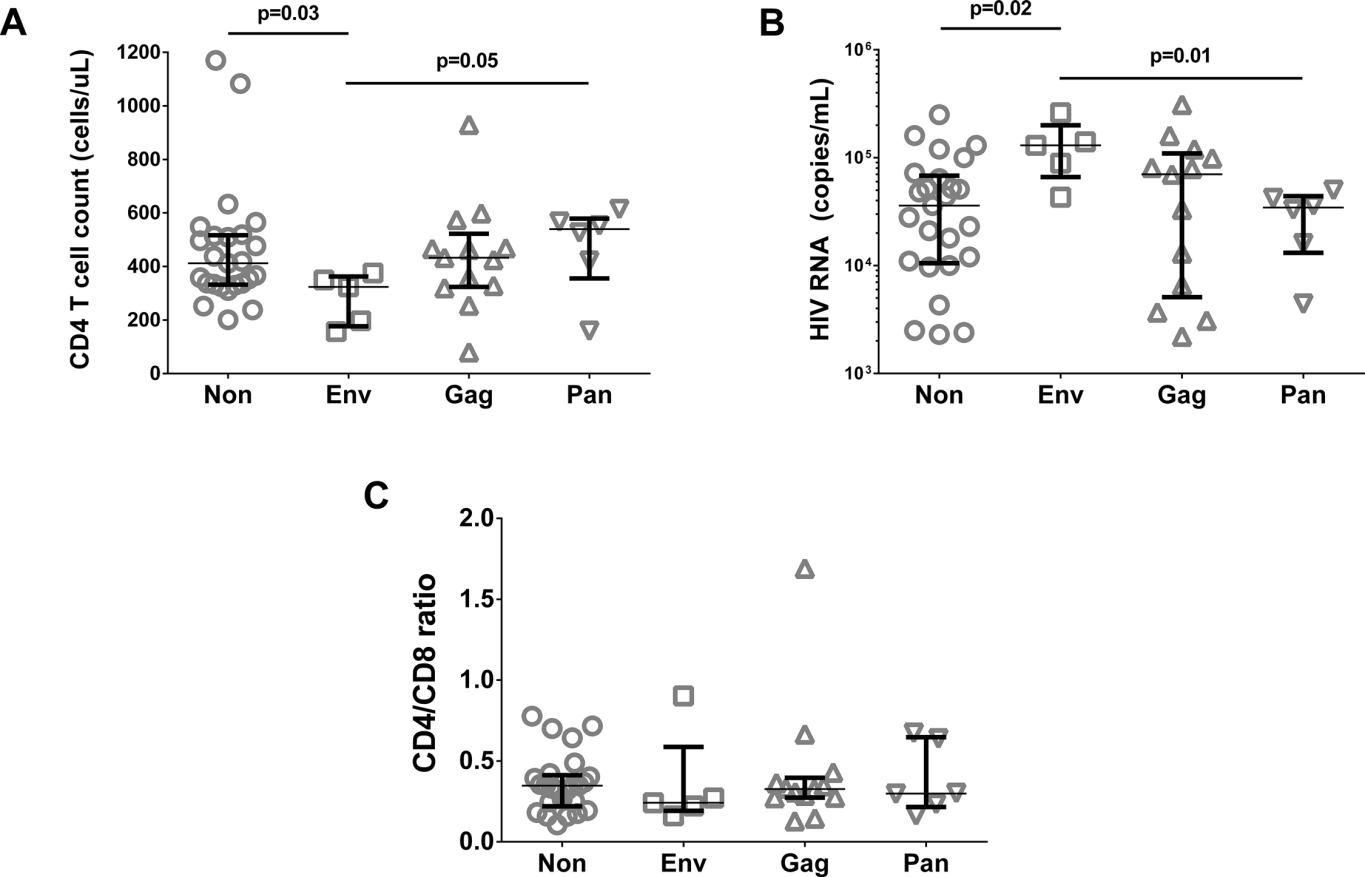
Lower CD4 counts and higher HIV viral loads in Env-regulators. (A) CD4 count, (B) Plasma HIV RNA and (C) CD4/CD8 ratio by regulator subgroup in the expanded cohort (n = 49). Median and upper/lower quartiles indicated. P-values derived from unpaired tests.

In order to confirm these observations, in particular the characteristics of more rapidly progressive HIV disease exhibited by Env-regulators, we reanalyzed CD8^+^ T cell Gag and Env FTR in another cross-sectionally sampled HIV cohort of 23 ART-naïve viremic patients. This supplementary cohort had similar clinical characteristics to the study cohort (CD4^+^ T cell count 425 (255–549) cells/uL; CD8^+^ T cell count 1222 (771–2019) cells/uL; plasma HIV RNA 52 000 (13 000–130 000) copies/mL; n.s.). When regulator subgroups were compared in the two cohorts combined (n = 49; 13 Gag-, 5 Env-, 6 Pan-, and 25 Non-regulators), we found that Env-regulators indeed had lower CD4 counts and higher HIV RNA than both Non- and Pan-regulators (see Fig 4).

### Concordant *proliferative* responses to HIV Gag and Env

As observed in previous studies, the proliferative responses of CD8^+^ T cells to Gag stimulation were greater than those induced by Env (p = 0.046, data not shown). In contrast to assessments of FTR, the CD8^+^ proliferative responses to the two antigens correlated (r = 0.65, p<0.001). The CD8^+^ T cell activation phenotype, as defined by co-expression of CD25 and HLA-DR, correlated closely with the proliferative responses in all culture conditions (r = 0.76–0.89, p<0.001).

Underscoring the complex relationship between proliferation and regulatory mechanisms, no significant correlations were found between antigen-specific proliferative responses and FTR mediated by any inhibitory pathway.

### Frequencies of activated Tregs following HIV antigen stimulation and signal blockade

**Fig 5:**
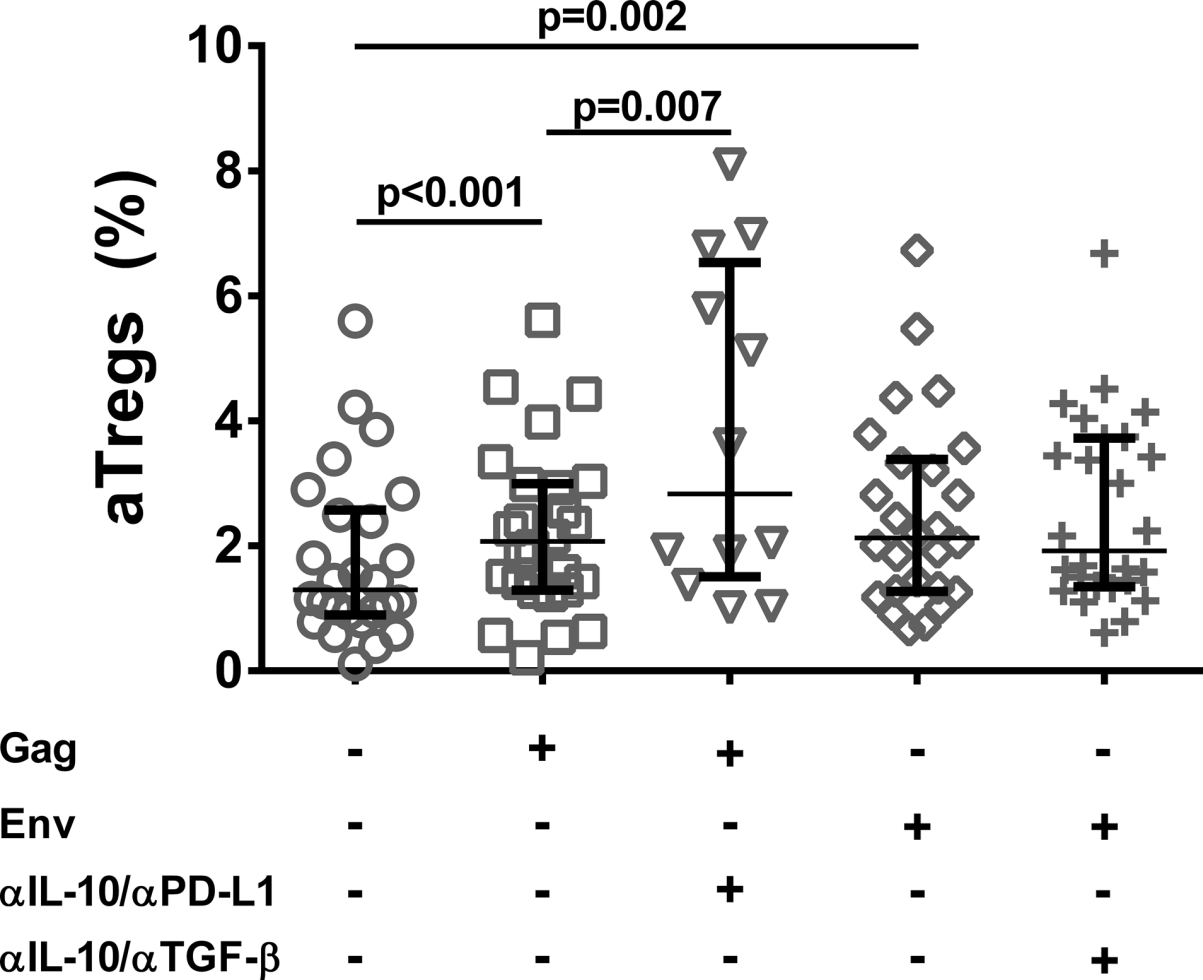
Increase in aTreg frequency after IL10/PD-1 dual blockade of Gag-stimulated cultures. Frequencies of activated regulatory T cells (CD25^hi^CD45RA^-^) in unstimulated cultures and in antigen-stimulated cultures with and without blocking antibodies. Median and upper/lower quartiles indicated. P-values derived from paired tests.

In the study cohort as a whole, the percentage of aTregs increased significantly after both Gag and Env stimulation *in vitro* (p<0.01, Fig 5). Dual blockade of IL-10/PD-L1 in Gag-stimulated cultures increased the aTreg fraction further (p = 0.007), whereas no such increase were observed in Env-stimulated cultures with dual blockade of IL-10/TGF-β.

## Discussion

The primary aim of this study was to explore and quantify antigen-specific functional T cell regulation by blocking defined inhibitory pathways in HIV antigen-stimulated cultures, with an ultimate goal of testing such parameters in the setting of functional HIV cure interventions. Our previous work to assess regulation with combined blockade of IL-10 and TGF-β was here expanded to include the PD-L1/PD-1 and CD160/HVEM pathways. We cross-sectionally tested samples from 26 HIV-infected, ART naïve patients, of who 14 (54%) had detectable FTR. Our main finding was substantial heterogeneity in the prevalence and magnitude of FTR between patients, inhibitory pathways as well as Gag and Env antigens, consistent with our previous observations [17]. Moreover, we have previously reported a similar variability in patients on ART, where this type of assay may help select patients for and/or predict the efficacy of therapeutic HIV vaccines [19].

In preliminary experiments, Antigen-specific, Cytokine-mediated regulation (R_AC_), as defined in our previous work [17] was also determined, taking into account downregulation of bystander proliferation in control cultures. We found minimal effects on background proliferation of blocking any of the assessed inhibitory pathways and thus a high concordance between RAC and FTR (data not shown).

We chose to use T cell proliferation after 5 days of culture as our primary read-out in assessing FTR. While interferon (IFN) γ secretion detected by ELISPOT or intracellular cytokine staining [25] and polyfunctionality [5,26] are commonly used measures of effector T cell responses to HIV peptides, these assays typically involve 6–18 hours of culture before analysis. We expected antigen-related regulation to develop more slowly, secondary to primary cell activation. In addition, the capacity of HIV-specific effector T cells to proliferate has been linked to delayed or non-progression of HIV infection [27,28], even at an epitope level [29]. Furthermore, proliferation has been linked to perforin expression by CD8^+^ T cells [28], essential for cytolysis of infected target cells. The high concordance between T cell proliferation and expression of the activation markers CD25 and HLA-DR was here additional evidence of T cell activation processes involving proinflammatory cytokines such as IFN-γ and IL-2 [30,31].

In our previous studies, we have quantified functional regulation by dual blockade of IL-10 and TGF-β [17–19]. This assay condition still proved most effective in revealing Env FTR in the current study cohort. By contrast, Gag FTR was best identified by combined IL-10 and PD-L1 blockade, in keeping with Porichis et al. [23]. Blockade of the PD-1/PD-L1 pathway is known to restore cytokine secretion and proliferative capacity of exhausted CD8^+^ T cells in HIV infection [32]. We found that the PD-1/PD-L1 pathway was involved in both Gag and Env FTR, but Gag-specific responses were more affected. As Gag-specific T cell responses in particular have been linked to control of HIV viremia [2,33], our data may lend further support to the use of anti-PD-1 antibodies in HIV immunotherapy [22]. Of note, the additive effect of concurrent PD-1 and IL-10 blockade was only apparent upon Gag stimulation.

Somewhat surprisingly, our assay neither detected Gag nor Env regulation by blocking IL-10 alone, as effects of IL-10Ra blockade on Gag p24-specific CD8^+^ T cell proliferation have been reported [34]. Notably, the monoclonal antibody used in our study (clone 23738) neutralises IL-10 bio-activity [17,35]. On the other hand, TGF-β alone mediated regulation in some patients, in accordance with Garba et al. [36]. The additive effect of blocking IL-10 and TGF-β in the same cultures may therefore suggest a potential recruitment of IL-10 dependent mechanisms during TGF-β blockade.

Regulatory effects of the CD160/HVEM pathway were detected in very few patients; two upon Gag stimulation and another patient upon Env stimulation. This contrasts with the results of Peretz et al. [37], who found significantly increased HIV-specific proliferation and cytokine secretion of CD8^+^ T cells during HVEM blockade. However, their experiments utilized peptides from HIV antigens other than Gag and Env.

Non-regulator patients with little or no detectable FTR by any of the two HIV peptide pools were clinically heterogeneous, with a wide range of CD4 counts and viral loads. This group was, however, also characterised by weak antigen-specific proliferative responses (i.e. unblocked), an observation which was confirmed in the expanded cohort (data not shown). Whether other regulatory mechanisms may be at play, or whether these HIV-specific T cell clones have in fact been deleted cannot be determined by our experiments. In a recent trial of therapeutic vaccination in another HIV-positive cohort, however, we have observed that patients who would have been denominated Non-regulators at study baseline may still develop FTR against the vaccine antigens [unpublished data].

Regulator patients displayed the same clinical heterogeneity as Non-regulators. While the apparent paucity of Env-regulators (approx. 10% in the expanded cohort) limits the conclusions which can be drawn, one may speculate that this group, exhibiting low HIV-specific proliferative capacity of CD8^+^ T cells, constitutes a patient phenotype more prone to clinical progression, in keeping with their higher HIV RNA levels compared to Non- and Pan-regulators. These findings are also in accordance with our previous work in another patient cohort, in which Env regulation was associated with accelerated CD4 loss [17]. Pan-regulators, meanwhile, had better T cell responses to both antigens, and induction of FTR may merely be a bystander effect of a strong proliferative drive in this group.

As could be expected, HIV antigen stimulation of PBMC increased the frequency of activated Tregs in culture. This potential for generation of Tregs after antigen exposure of CD4^+^ T cells is well established [38]. Interestingly, we observed a further increase in aTreg frequencies in Gag-stimulated cultures with dual blockade of IL-10 and PD-L1, but not in Env-stimulated cultures with IL-10/TGF-β blockade. As both TGF-β and the PD-1/PD-L1 axis has been implicated in the induction of Tregs [39–42], the mechanisms contributing to these antigen-dependent differences are unclear, and cannot be elucidated by our straightforward *in vitro* assay.

The increase in ART coverage and potential shift of focus to functional cure strategies in HIV infection encourage the extension of our study to ART-treated patients with suppressed viremia. The limited number of patients in this study as well as the substantial heterogeneity in T cell proliferation, FTR and HIV antigen dependency restrict the conclusions we can draw. Furthermore, while the regulation profiles of CD8^+^ T cells as described here may have clinical significance, a cross-sectional approach cannot ascertain whether they represent a cause or consequence of progression.

In conclusion, assuming that enhanced proliferation of CD8^+^ T cells by blockade of key inhibitory pathways represents functional regulation *in vivo*, our data confirm that FTR of HIV-specific CTL responses is common in HIV-infected patients. Although our data indicate that Gag and Env antigens are differentially regulated in patients, we nevertheless found combinations of inhibitory pathway blockers that identified all patients with Gag and Env FTR, respectively. Taken together, our data support the notion that interventions to improve HIV-specific immunity should be individually tailored and that assessments of FTR should be further explored in clinical trials.

## Supporting Information

S1 Fig (TIF)
