# Supplementary figures and images for: Regulation of Gag- and Env-Specific CD8+ T Cell Responses in ART-Naïve HIV-Infected Patients: Potential Implications for Individualized Immunotherapy
Source: PLoS One. 2016 Apr 29;11(4):e0153849. doi: 10.1371/journal.pone.0153849 (PMC4851414; doi:10.1371/journal.pone.0153849)

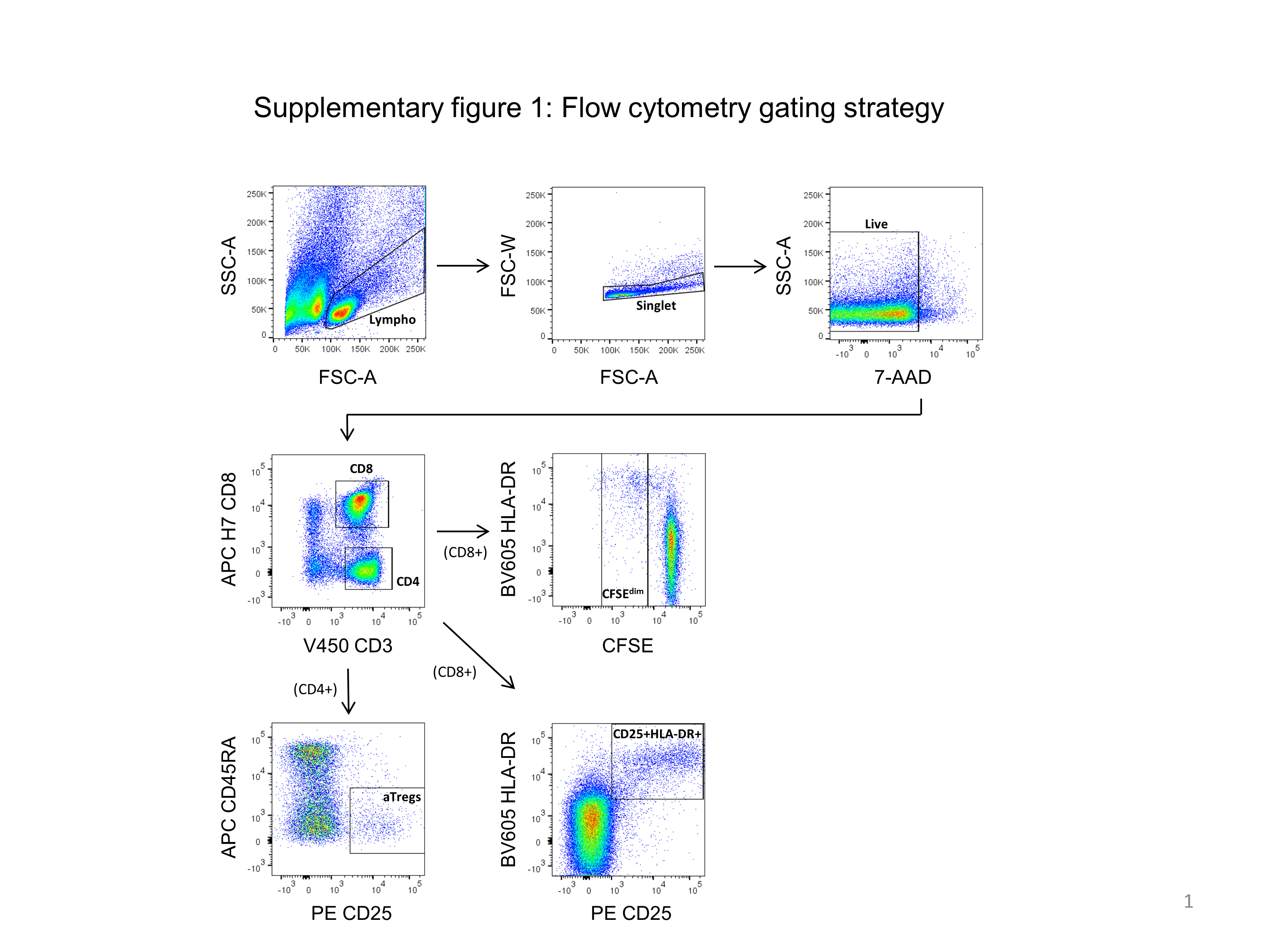

Supplement: S1 Fig — (TIF) [file pone.0153849.s001.tif]
